# Structural and biochemical characterization of DAXX-ATRX interaction

**DOI:** 10.1007/s13238-017-0463-x

**Published:** 2017-09-05

**Authors:** Zhuang Li, Dan Zhao, Bin Xiang, Haitao Li

**Affiliations:** 1 College of Life Sciences, Peking University, Beijing, 100871 China; 2 MOE Key Laboratory of Protein Sciences, Tsinghua-Peking Joint Center for Life Sciences, Beijing Advanced Innovation Center for Structural Biology, Department of Basic Medical Sciences, School of Medicine, Tsinghua University, Beijing, 100084 China

**Dear Editor,**

Epigenetic factors, acting as co-activators/repressors in transcription, regulate diverse cellular activities ranging from cell growth and differentiation to host defense and immune response. Covalent histone or DNA modifications, histone variants, ATP-dependent chromatin remodeling, and non-coding RNA are major mechanisms underlying epigenetic regulation. In particular, ATP-dependent chromatin remodeling complexes and histone chaperones are key factors that regulate chromatin dynamics.

Death domain-associated protein (DAXX), originally identified as a Fas death receptor binding protein during apoptosis, was characterized as an H3.3-specific histone chaperone to promote replication-independent deposition of H3.3 (Drane et al., 2010; Lewis et al., 2010). DAXX harbors an N-terminal DAXX helical bundle (DHB), a histone-binding domain (HBD) in the middle, and a C-terminal intrinsically disordered region (Fig. 1A). The DHB domain reportedly recognizes a consensus motif of different DAXX binding partners (Escobar-Cabrera et al., 2010). The HBD domain harbors histone chaperone activity and specifically envelops the histone H3.3-H4 dimer for H3.3-specific recognition (Elsasser et al., 2012; Liu et al., 2012).

**Figure 1:**
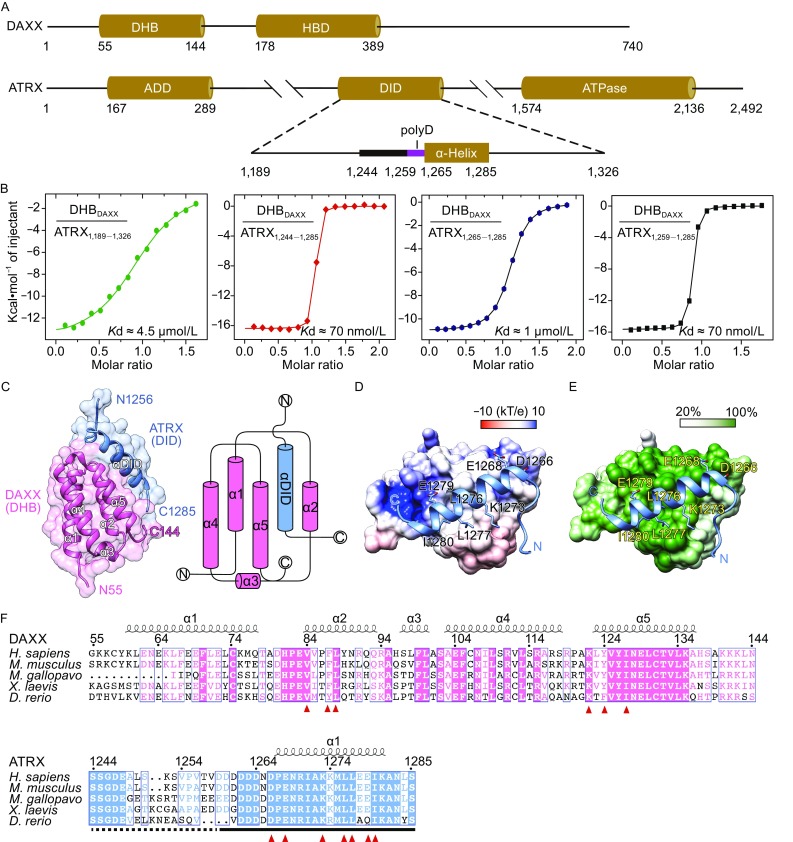
**Overall structure of DAXX-ATRX mini-complex**. (A) The domain architecture of DAXX and ATRX. (B) ITC fitting curves of the DHB domain of DAXX (DHB_DAXX_) titrated with ATRX_1,189–1,326_, ATRX_1,244–1,285_, ATRX_1,265–1,285_, and ATRX_1,259–1,285_. (C) Surface and topological representation of DAXX-ATRX mini-complex. DHB_DAXX_ and ATRX_1,256–1,285_ peptide are coded pink and blue, respectively. (D) Electrostatic potential surface view of the DAXX-ATRX mini-complex. DAXX is colored as a spectrum of its surface electrostatic potential ranging from blue (10 kT/e) to red (−10 kT/e). (E) Conservation mapping surface view of the DAXX-ATRX mini-complex. DAXX is colored based on the surface conservation score among orthologs aligned in panel (F). (F) Sequence alignment of DAXX and ATRX orthologs in vertebrates. Red triangles, key residues participating in DAXX-ATRX interaction. Dashed lines, invisible sequence in the crystal structure

ATRX was discovered to map the genetic mutations that lead to the ɑ-thalassemia, mental retardation, X-linked (ATRX) syndrome (Gibbons et al., 1995). This ATP-dependent chromatin remodeling protein contains an N-terminal ATRX-DNMT3-DNMT3L (ADD) domain, partner-binding regions in the middle and a C-terminal ATPase domain (Fig. 1A). The ADD domain functions as a reader module that recognizes histone H3 “K4me0-K9me3” methylation pattern and tolerates additional H3S10 phosphorylation to facilitate heterochromatin targeting of ATRX (Iwase et al., 2011; Noh et al., 2015). Regions for binding macroH2A, EZH2, HP1, DAXX, and MeCP2 have been identified in ATRX. These interactions contribute to the cellular localization and activities of ATRX (Ratnakumar and Bernstein, 2013).

ATRX physically interacts with DAXX to promote the incorporation of H3.3 into telomeric, pericentromeric, and other repetitive DNA regions (Drane et al., 2010; Goldberg et al., 2010; Lewis et al., 2010). Under conditions of DNA hypomethylation, DAXX-ATRX complex is essential to promote H3K9 trimethylation around the tandem repetitive elements, thus safeguarding the genome stability in embryonic stem cells (He et al., 2015). The interaction regions between DAXX and ATRX have been mapped to the DHB domain of DAXX (DHB_DAXX_, aa 55–144) and the DAXX interaction domain of ATRX (DID_ATRX_, aa 1,189–1,326) (Fig. 1A) (Tang et al., 2004).

To further map the minimal region of DID_ATRX_ essential for DAXX interaction, we generated a series of DID_ATRX_ truncations based on secondary structural analysis, and performed isothermal titration calorimetry (ITC) studies (Fig. 1B). We measured a dissociation constant (*K*_D_) of 4.5 μmol/L between full length DID_ATRX_ (aa 1,189–1,326) and DHB_DAXX_. Strikingly, we detected a binding *K*_D_ of 70 nmol/L between a 42-residue segment of DID_ATRX_ and DHB_DAXX_. This segment is characteristic of a loop-helix motif and spans residues 1,244–1,285 of ATRX (Fig. 1A). The observed ~64-fold enhancement of affinity underscores the binding potency of ATRX_1,244–1,285_, and suggests an auto-inhibitory role of the ATRX_1,244–1,285_-flanking sequence. The binding *K*_D_ dropped to 1 μmol/L when the α-helix frame only (α_DID_, ATRX_1,265–1,285_) was used for ITC titration, stressing the role of loop ATRX_1,244–1,264_ in promoting DAXX-ATRX interaction.

We next co-expressed, purified, crystallized the complex of DHB_DAXX_-ATRX_1,244–1,285_, and solved the crystal structure at 1.58 Å by zinc single wavelength anomalous dispersion (Table S1). Full length DHB_DAXX_ and residues 1,256–1,285 of ATRX were modelled based on the electron density map (Fig. S1). DHB_DAXX_ features a core four-helical bundle and ATRX_1,256–1,285_ adopts a loop-helix fold that targets the “α2-α5” surface of DHB_DAXX_ (Fig. 1C). In the crystal, the modelled DHB_DAXX_-ATRX_1,256–1,285_ complex forms a “head-to-head” dimer organized around the α3 linker helix of DHB_DAXX_ (Fig. S2A). Despite extensive interactions, our SEC-MALS (size exclusion chromatography followed by multi-angle light scattering) analysis suggested that the DHB_DAXX_-ATRX_1,244–1,285_ complex exists as a monomer in solution and the observed dimer formation is likely due to crystal packing (Fig. S2B and S2C). The free and complex states of DHB_DAXX_ are well superimposable without clear conformational change (Fig. S2D), suggesting that the ATRX binding surface of DHB_DAXX_ is largely preformed. Since only ATRX_1,259–1,285_ is visible in the complex structure, we next tested if this short motif is sufficient for DAXX binding. A binding *K*_D_ of 70 nmol/L was measured between ATRX_1,259–1,285_ and DHB_DAXX_ (Fig. 1B), which is nearly the same as ATRX_1,244–1,285_. Thus, our structural studies further defined a minimum ATRX motif of only 27 residues for high affinity DAXX interaction. Electrostatic potential analysis revealed that ATRX_1,259–1,285_ covers an elongated surface that is hydrophobic in the center flanked by electrostatic positive patches (Fig. 1D). Upon complex formation, we calculated a buried solvent accessible area of 1,111 Å^2^, which accounts for ~18% of the total solvent accessible area of DHB_DAXX_. Residues constituting the ATRX binding surface of DAXX are highly conserved among vertebrate species ranging from zebrafish to human (Fig. 1E and 1F), highlighting their functional consensus.

ATRX_1,259–1,285_ engages extensive interactions with DHB_DAXX_ to form a compact five-helical bundle (Figs. 1C and 2A). These interactions can be classified into three groups including a central hydrophobic core and two flanking polar clusters. The central hydrophobic core is composed of V84, F87, L88, Y124 and I127 of DAXX and L1276, L1277, I1280, K1273 (via its hydrocarbon portion) of ATRX (Fig. 2B). Human ATRX_1,259–1,285_ is characteristic of an N-terminal polyD loop (_1,259_DDDDDD_1,264_) (Fig. 1F). Deletion of the polyD loop led to an affinity drop from 70 nmol/L to 1 μmol/L (Fig. 1B), stressing its binding contribution. Notably, the polyD loop is structurally organized by zinc coordination (involving residues D1259, D1261, D1264, D1262) and intra-ion pair formation (D1263-K1273) (Fig. 2C). This stabilized structural unit of polyD loop further interacts with DHB_DAXX_ via a network of water-mediated hydrogen-bonding interactions (Fig. 2C). Next to the polyD loop is a positive surface patch formed by residues R111, R115, R119 and K122 of DAXX (Fig. 2A), which may electrostatically facilitate DAXX-ATRX interaction. Particularly, K122 directly pairs with E1268 and D1266 of ATRX to facilitate binding (Fig. 2D). The other set of polar cluster is formed around the C-terminal part of the loop-helix motif of ATRX. In particular, these interactions include a unique zinc finger coordination involving residues E1279 of ATRX and C131 of DAXX, as well as ion pairs between R91 of DAAX and S1285, E1279 of ATRX (Fig. 2D).

**Figure 2:**
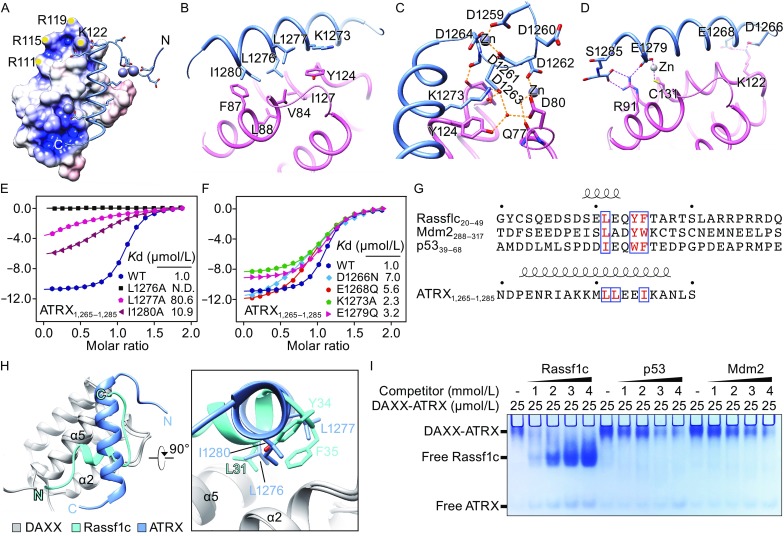
**Interaction details, mutagenesis study and competition assay**. (A) Positioning of ATRX loop-helix motif on DHB_DAXX_. DHB_DAXX_ is in electrostatic potential surface view as defined in Fig. 1D. ATRX is in ribbon representation with key residues in stick view. Yellow dots denote the positions of four annotated basic residues. Grey spheres, zinc ions. (B) Details of hydrophobic contacts between DHB_DAXX_ (pink) and ATRX α-helix (blue). Key residues are shown in stick view. (C) Details of hydrophilic contact involving the polyD loop of ATRX (blue). Gray spheres, zinc ions; small red balls, water molecules; dashed lines, hydrogen bonds. (D) Details of hydrophilic interactions involving ATRX α-helix (blue). (E and F) ITC fitting curves of DHB_DAXX_ titrated with denoted ATRX_1,265–1,285_ mutant peptides. WT, wild type peptide control. (G) Sequence alignment among Rassf1c (20–49), Mdm2 (288–317), and p53 (39–68), and ATRX (1,265–1,285) peptides. Blue boxes highlight key hydrophobic residues of the consensus motif. (H) Structural superimposition of DHB_DAXX_-Rassf1c with DHB_DAXX_-ATRX. DHB_DAXX_, Rassf1c, and ATRX peptides are colored gray, cyan and blue, respectively. Right, “close-up” view of the central hydrophobic core. Key hydrophobic residues are depicted as sticks. (I) Electrophoretic mobility shift assay to detect the disassociation of preformed DAXX-ATRX complex upon incubation with denoted competitor peptides. Protein bands are detected by Coomassie blue staining. Only DAXX-ATRX, free Rassf1c, and free ATRX peptides can enter the gel and are labelled accordingly. Frame information are as the follows: DAXX, 55–144; ATRX, 1,244–1,285; Rassf1c, 20–44; p53, 39–63; Mdm2, 293–317

Next, we synthesized mutant ATRX peptides in the frame of 1,265–1,285, and performed ITC study to validate the observed interactions. As expected, L1276A completely disrupt DBH_DAXX_ binding, while the binding *K*_D_ dropped from 1 μmol/L to 80.6 μmol/L for L1277A and 10.9 μmol/L for I1280A (Fig. 2E). Consistent with our structural analysis, the *K*_D_ values dropped 2.3- to 7-fold between DBH_DAXX_ and different ATRX polar residue mutants such as D1266N, E1268Q, K1273A and E1279Q (Fig. 2F). The more pronounced binding loss in the cases of L1276A, L1277A and I1280A underscores a critical role of the hydrophobic core to nucleate DAXX-ATRX interaction.

Besides ATRX, DHB_DAXX_ also interacts with other partners, such as Rassf1c, Mdm2, and p53 that share a consensus motif (Fig. 2G). The NMR solution structure of DHB_DAXX_-Rassf1c complex has been reported (Escobar-Cabrera et al., 2010). Structural alignment revealed that ATRX and Rassf1c target the same “α2-α5” surface of DHB_DAXX_ (Fig. 2H). Interestingly, the ATRX and Rassf1c peptides adopt distinct conformations for DHB_DAXX_ targeting. As shown in Fig. 2H, the helix elements of ATRX and Rassf1c are almost perpendicular to each other. The most conserved binding feature is the central hydrophobic core that is contributed by residues L1276, L1277, I1280 in the case of ATRX and residues L31, Y34, F35 in the case of Rassf1c (Fig. 2H). Notably, a leucine residue is structurally conserved in both ATRX (L1276) and Rassf1c (L31), and is anchored at the central hydrophobic pocket of DHB_DAXX_ to nucleate binding.

DAXX and ATRX form a complex in promyelocytic leukemia (PML) nuclear bodies and heterochromatin to regulate gene activity and chromatin structure. DAXX recruits Rassf1C into PML nuclear bodies and releases it when DAXX is degraded upon DNA damage (Kitagawa et al., 2006). DAXX interacts with the E3 ligase Mdm2 in PML nuclear bodies and prevents its self-ubiquitination, thus mediating the proteolytic degradation of p53 (Tang et al., 2006). DAXX also interacts with p53 to regulate its activity by competitive interaction with PML (Kim et al., 2003). Given the shared binding mode and cellular localization of the abovementioned DAXX partners, it is interesting to explore their competitive feature. To this end, we performed peptide competition assays based on electrophoretic mobility shift assay. The preformed DHB_DAXX_-ATRX_1,244–1,285_ complex was incubated with competitor peptides (Rassf1c_20–44_, p53_39–63_ and Mdm2_293–317_) of different concentration, and then subjected to native-PAGE analysis. As shown in Fig. 2I, the DAXX-ATRX complex was gradually disrupted by the competitor peptides in a concentration-dependent manner, among which the Rassf1c peptide displayed the strongest competitor activity, followed by p53 and Mdm2 peptides. These results indicate that ATRX, Rassf1c, Mdm2, and p53 bind to DAXX in a mutually exclusive manner and they likely compete for DAXX interaction under regulated conditions, thus suggesting a “partner switch” mechanism in DAXX biology.

## Electronic supplementary material

Below is the link to the electronic supplementary material.

Supplementary material 1 (PDF 618 kb)
