# Supplementary material for: Structural and biochemical characterization of DAXX-ATRX interaction
Source: Protein Cell. 2017 Sep 5;8(10):762–6. doi: 10.1007/s13238-017-0463-x (PMC5636754; doi:10.1007/s13238-017-0463-x)
Supplement: Supplementary file 1 — Supplementary material 1 (PDF 618 kb) [file 13238_2017_463_MOESM1_ESM.pdf]

Supplementary Information

Structural and biochemical characterization of DAXX-ATRX interaction

Zhuang Li,[1,2] Dan Zhao,[2] Bin Xiang,[1,2] and Haitao Li[2,*]

[1]College of Life Sciences, Peking University, Beijing 100871, P.R. China

[2]MOE Key Laboratory of Protein Sciences, Tsinghua-Peking Joint Center for Life Sciences, Beijing Advanced Innovation Center for Structural Biology, Department of Basic Medical Sciences, School of Medicine, Tsinghua University, Beijing 100084, P.R. China

**Correspondence: lht@tsinghua.edu.cn (H.L.)*

To whom correspondence should be addressed:

Haitao Li

Medical Science Building C228

Tsinghua University

Beijing 100084, P.R.China

Tel: (+) 86-10-62771392

E-mail: lht@tsinghua.edu.cn

Running title: Molecular basis of DAXX-ATRX interaction

Keywords: DAXX, ATRX, chromatin remodeling, histone chaperone, transcription

EXPERIMENTAL PROCEDURES

Molecular cloning, protein expression and purification

The DAXX-ATRX mini-complex for crystallization was produced using a co-expression strategy. The truncated DID domain ($ATRX_{1244\text{-}1285}$) of ATRX (UniProt ID: P46100) were ligated into the first multiple cloning site (MCS) of the pRSFD-SUMO vector (modified from pRSFDuet-1, Novagen) with an N-terminal 6xHis-SUMO tag, and the DHB domain ($DAXX_{55\text{-}144}$) of DAXX (UniProt ID: Q9UER7) was ligated into the second MCS of pRSFD-SUMO without any tag. The transformed BL21 (DE3) cells (Invitrogen) were grown at 37°C in LB medium to reach an OD_{600nm} = 0.6-0.8, and then induced with 0.2 mM IPTG at 18°C overnight for recombinant protein expression. Centrifugation-collected cells were suspended in lysis buffer containing 500 mM NaCl, 20 mM imidazole and 50 mM Tris, pH 7.5, and then lysed by an EmulsiFlex-C3 high pressure homogenizer (AVESTIN). The cell lysates were further clarified by centrifugation and the 6xHis-SUMO-$ATRX_{1244\text{-}1285}$-$DAXX_{55\text{-}144}$ mini-complex in supernatant was affinity purified through a HisTrap HP column (GE Healthcare). The 6xHis-SUMO tag was cleaved by the SUMO protease, Ulp1. After removal of imidazole from the digestion mixture by desalting column, the 6xHis-SUMO tag was affinity eliminated by an additional HisTrap HP column. The flow-through was then pooled, concentrated, and applied to a Superdex 75 10/300 GL (GE Healthcare) gel filtration column under the elution buffer containing 100 mM NaCl, 10 mM Tris, pH 7.5. The resultant peak was then pooled and concentrated to about 15 mg/ml for future use.

Free DHB domain of DAXX was constructed into the pGood6p vector (modified from pGEX-6p-1, GE Healthcare) with an N-terminal GST-tag. The recombinant protein was expressed in BL21 (DE3) cells. Cells were grown at 37°C and induced with 0.2 mM IPTG at 18°C overnight for protein expression and lysed in lysis buffer containing 500 mM NaCl, 50 mM Tris, pH 7.5. Procedures for protein purification include GST affinity purification, human rhinovirus 3C protease digestion, S cation exchange, and gel filtration. The purified DHB_{DAXX} sample containing an N-terminal “GPLGS” uncleavable tag sequence was stored in ITC buffer containing 100 mM NaCl, 20 mM HEPES, pH 7.5 for future use.

Full length DID domain of ATRX ($ATRX_{1189\text{-}1326}$) was constructed into the pET28-b vector and expressed in BL21(DE3) cells. Cells were grown at 37°C and induced with 0.2 mM IPTG at 18°C overnight for protein expression and lysed in lysis buffer containing 500 mM NaCl, 20

mM imidazole, and 50 mM Tris, pH 7.5. Procedures for protein purification include Ni-NTA affinity purification, thrombin digestion, Q anion exchange, and gel filtration. The purified DID_{ATRX} sample containing an N-terminal "GSHM" uncleavable tag sequence was stored in ITC buffer containing 100 mM NaCl, 20 mM HEPES, pH 7.5 for future use.

Crystallization and structure determination

Crystals were grown by the sitting-drop vapor-diffusion method at 18°C by mixing 1 μL of the DAXX-ATRX mini-complex (15 mg/ml) with 1 μL of the reservoir solution. Crystals were obtained under the reservoir solution: 13% ethanol, 100 mM MES, pH 6.0, and 100 mM zinc acetate. Crystals were flash-frozen under cryoprotectant solution composed of the reservoir solution supplemented with 20% (v/v) glycerol. Diffraction data were collected under cryo-conditions at 0.9778 Å at the Shanghai Synchrotron Radiation Facility (SSRF) beamline BL19U1 of National Center for Protein Science, Shanghai. Due to the high resolution of the data set (1.58 Å), decent zinc anomalous scattering signal can be detected for phasing. Data reduction, phasing, and initial model building were done with HKL3000 suite (Minor et al., 2006). Further model building and refinement were done with COOT and PHENIX (Adams et al., 2010; Emsley and Cowtan, 2004). Detailed data processing and structural refinement statistics are summarized in table S1. Structural figures were created using UCSF Chimera (Pettersen et al., 2004) or PyMOL (https://pymol.org).

Isothermal titration calorimetry (ITC)

Calorimetric experiments were conducted at 25°C with a MicroCal iTC200 instrument (GE Healthcare). The DHB_{DAXX} and full length DID_{ATRX} samples were prepared in the buffer containing 100 mM NaCl and 20 mM HEPES, pH 7.5. Protein concentration was determined by absorbance spectroscopy at 280 nm. The DID_{ATRX} truncation peptides were chemically synthesized by Scilight Biotechnology LLC (Beijing, China), and were quantified by weighing on a large scale. Acquired calorimetric titration data were analyzed using Origin 7.0 (GE Healthcare) using the "One Set of Binding Sites" fitting model. All the ITC experiments in this study used DID_{ATRX} as the "ligand" in the syringe and the DHB_{DAXX} in the sample cell.

Peptide competition electrophoretic mobility shift assay (EMSA)

In the peptide competition EMSA assay, purified protein complex stock of DAXX-ATRX as described above was prepared in the reaction buffer (100 mM NaCl and 20 mM HEPES, pH 7.5) at concentration of about 0.5 mM. The competitor peptides (Scilight Biotechnology LLC), Rassf1c$_{20-44}$, p53$_{39-63}$ and Mdm2$_{293-317}$, were dissolved in reaction buffer at a concentration of 4 mM. For each competition reaction, DAXX-ATRX complex was mixed with competitor peptide with or without reaction buffer to a total volume of 20 µL. The mixture was incubated on ice for 1 hour before subjected to native-PAGE (polyacrylamide gel electrophoresis) analysis. In an optimized native-PAGE system, the Tris buffer at pH 7.5 was used to prepare the polyacrylamide gel and Tris-MOPS, pH 8.0, was used as the running buffer.

Supplementary Table S1

	$DAXX_{55-144}$-$ATRX_{1244-1285}$
Data collection	
Wavelength (Å)	0.9778
Space group	$P2_12_12_1$
Cell dimensions	
a, *b*, *c* (Å)	53.4, 56.2, 102.9
α, β, γ (°)	90, 90, 90
Resolution (Å)	50 -1.58 (1.61 - 1.58)*
R_{merge} (%)	9.4 (55.0)$^{\$}$
$I / \sigma I$	19.8 (2.5)
Completeness (%)	99.8 (95.7)
Redundancy	6.8 (6.2)
Refinement (F>0)	
Resolution (Å)	50 – 1.58
No. of reflections (test set)	42638 (4060)
R_{work} / R_{free} (%)	15.7 / 17.9
No. of atoms	
Proteins	1882
Ligands	41
Solvent	409
B-factors (Å^2)	
Proteins	19.2
Ligands	24.9
Solvent	33.1
R.m.s. deviations	
Bond lengths (Å)	0.006
Bond angles (°)	0.786

* Values in parentheses are for outmost shell

$ This data set contain anomalous signal

Supplementary Figure S1

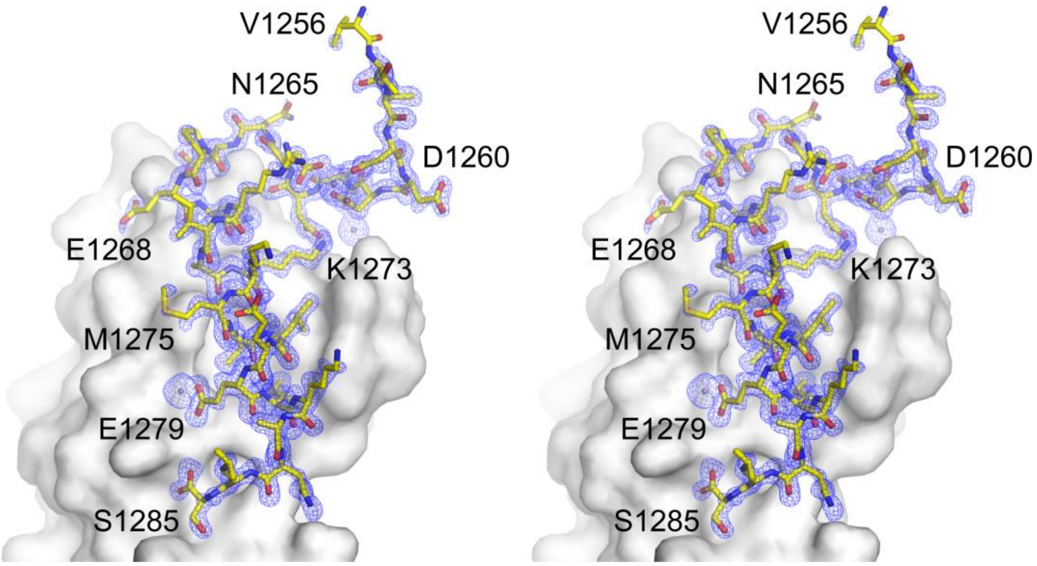

Fig. S1 Electron density map around the modeled ATRX$_{1256\text{-}1285}$ loop-helix motif.

DAXX DHB domain is shown in surface view. Modelled ATRX$_{1256\text{-}1285}$ is shown in stick view. Blue mesh, Fo-Fc omit map contoured at 2.5 σ level. ATRX$_{1256\text{-}1285}$ and three zinc ions (small grey ball) are omitted for map calculation. Representative residues of ATRX$_{1256\text{-}1285}$ are labelled. Residue S1285 contains a C-terminal carboxylate group as it is the last residue of the co-expressed ATRX$_{1244\text{-}1285}$ peptide for crystallization. Figure is prepared in stereoview.

Supplementary Figure S2

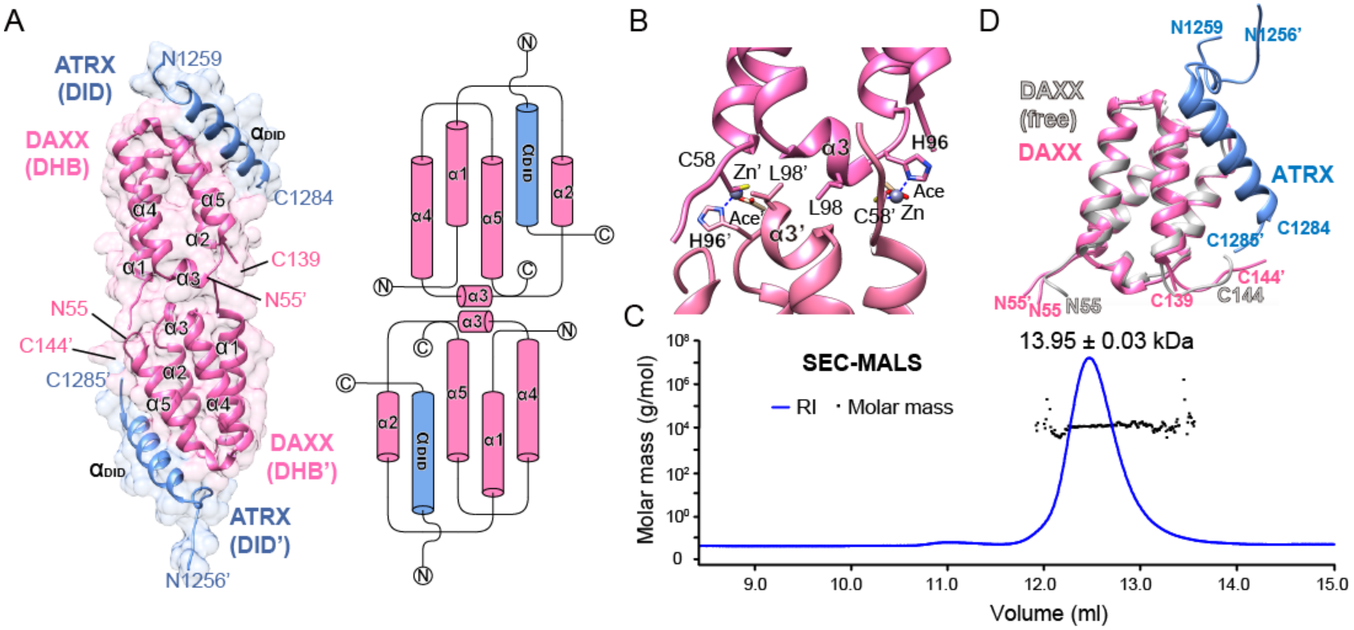

Fig. S2 The DHB$_{DAXX}$-ATRX$_{1244\text{-}1285}$ complex forms a dimer in crystal and behaves as a monomer in solution.

(A) Overall structure and topological representations of the DHB$_{DAXX}$-ATRX$_{1244\text{-}1285}$ dimer in one asymmetric unit. α1，α2，α3，α4，α5 denote helices of the DHB domain, and the α$_{DID}$ denotes the α-helix of ATRX peptide. (B) Interaction details at the dimer interface around the α3 linker helix. Blue dashed-lines denote the zinc-coordination bonds. Gray spheres, zinc ions. (C) Size exclusion chromatography followed by multi-angle light scattering analysis (SEC-MALS) to examine the molar mass of DHB$_{DAXX}$-ATRX in solution. The black dots denote the molar mass of proteins and the blue chromatography denotes the RI. (D) Superposition of two DHB$_{DAXX}$-ATRX$_{1244\text{-}1285}$ monomers and free DHB$_{DAXX}$.

Reference

Adams, P.D., Afonine, P.V., Bunkoczi, G., Chen, V.B., Davis, I.W., Echols, N., Headd, J.J., Hung, L.W., Kapral, G.J., Grosse-Kunstleve, R.W., *et al.* (2010). PHENIX: a comprehensive Python-based system for macromolecular structure solution. Acta Crystallogr D Biol Crystallogr *66*, 213-221.

Emsley, P., and Cowtan, K. (2004). Coot: model-building tools for molecular graphics. Acta Crystallogr D Biol Crystallogr *60*, 2126-2132.

Minor, W., Cymborowski, M., Otwinowski, Z., and Chruszcz, M. (2006). HKL-3000: the integration of data reduction and structure solution--from diffraction images to an initial model in minutes. Acta Crystallogr D Biol Crystallogr *62*, 859-866.

Pettersen, E.F., Goddard, T.D., Huang, C.C., Couch, G.S., Greenblatt, D.M., Meng, E.C., and Ferrin, T.E. (2004). UCSF Chimera--a visualization system for exploratory research and analysis. J Comput Chem *25*, 1605-1612.
